# Association analysis of vitamin D receptor gene polymorphisms in North England population with Type 2 diabetes mellitus

**DOI:** 10.4314/ahs.v21i1.3

**Published:** 2021-03

**Authors:** Naila Abdul Sattar, Sumera Shaheen, Fatma Hussain, Amer Jamil

**Affiliations:** 1 Department of Bio-Chemistry, Government College Women University, Faisalabad-38000; 2 Department of Bio-Chemistry, University of Agriculture, Faisalabad-38000

**Keywords:** T2DM, DNA Sequencing, Polymerase Chain Reaction-Restriction Fragment Length Polymorphism (PCR-RFLP), Single nucleotide Polymorphism (SNP), VDR

## Abstract

**Background:**

Numerous diabetes susceptibility loci, include a region consisting vitamin D receptor gene found in chromosome 12q, have been known using genome wide screens.

**Aim:**

The aim of present study is to probe the relationship between polymorphism of vitamin D receptor gene (single nucleotide polymorphisms) and type 2 diabetes mellitus (T2DM). Five hundred T2DM patients and 200 healthy subjects with normal HbA1c (≤ 5.0 %), fasting blood sugar (≤ 120 mg/dL) and random blood sugar (≤ 140 mg/dL) were enrolled.

**Metholodgy:**

The genotypes were found by polymerase chain reaction restriction fragment length polymorphism and DNA sequencing.

**Results:**

revealed that no considerable differences in frequencies of genotype and allele of the Bsm I and Fok I polymorphisms between healthy and patients in the North England (For Fok I: OR = 1.11, 95% CI: 0.72–1.12; for Bsm I: OR = 1.35, 95% CI: 0.79–1.98).

**Conclusion:**

It is recommended that both following polymorphisms of vitamin D receptor gene may not considerably add to the progression of T2DM in the North England.

## Introduction

The most ubiquitous form of diabetes is T2DM. More than 90% of all diagnosed diabetic cases belong to this type, affecting 246 million people worldwide. It is characterized by insulin resistance and beta cell dysfunction and is one of the leading causes of death and disability^1^,^2^. Perhaps there are a number of different causes of T2DM, though exact etiologies are still not known. Combination of genetic and environmental factors that contribute to T2DM onset are life style, dietary habit, BMI, hypovitaminosis D and family history^3^,^4^. The bioavailability of vitamin D3 may be good biomarker for the association of vitamin D to bone mineral density, nephron osteodystrophy and T2DM^5^,^6^. There are at least 64 common genetic variants that are strongly associated with T2DM. Variations in the gene sequences such as single nucleotide polymorphisms (SNP) explain the individual differences in traits like disease susceptibility and response to treatment^7^. Candidate genes for T2DM risk present in specific genome parts are classified as those involved in disease onset, associated pathways and functions^8^. VDR gene is present on chromosome 12q12-q14 9, which mediates vitamin D action as it binds to vitamin D response elements (VDRE)^10^,^11^. A number of VDR variants have been observed in early 1990s; ApaI, BsmI, EcoRV, TaqI, Tru9I, FokI and CDX2. Recently, four contiguous restriction fragment length polymorphisms for BsmI, TaqI, FokI and ApaI have been found associated to T2DM 12,13. However, studies on association between VDR genetic polymorphisms and risk of T2DM in different ethnic groups is notconclusive. Comprehensive understanding of VDR genetic polymorphisms would help uncover their impact in T2DM. As the progress in identification of VDR genetic variants predisposing to T2DM in following population has been limited, therefore, present research was conducted with the aim to examine this candidate gene in T2DM patients.

## Materials and methods

A total of 500 related T2DM patients were recruited from local North England hospital, Bristol University from July 2014 to March 2015. The patients (50% males and 50% females) with average age of 40.5 year old showed typical clinical symptom of T2DM. While, total of 200 controls with same ratio of male and female was selected. Written informed consent was obtained from all the subjects, and the study was performed with the approval of the ethics committee of the Research Medical Council, Bristol, UK.

### Genotyping

Genomic DNA was extracted from the collected blood. Salting out method for DNA extraction was implied using proteinase K, by peptide hydrolysis and a saturated NaCl solution for cellular dehydration and protein precipitation. Genomic DNA was recovered by standard salt and ethanol precipitation^14^. VDR polymorphisms were identified by using polymerase chain reaction-restriction fragment length polymorphism (PCR-RFLP) analysis. Experimental conditions including primer sequences, reaction conditions, restriction enzymes used and length of resulting PCR products are shown (Table 2). 15 To confirm the accuracy of genotyping, partial samples were examined by DNA sequencing.

**Table 2:** VDR primers sequences and reaction conditions for genotyping polymorphisms

VDR Polymorphism	Primer	PCR annealing temperature (°C)	Product size (bp)	Incubation time (minutes)
***Fok*I**	**F:CCCTGGCACTGACTCTGCTC** **R:GGAAACACCTTGCTTCTTCTCC**	**60**	70, 197, 267	60
***Bsm*I**	**F:AGTGTGCAGGCGATTCGTAG** **R:ATAGGCAGAACCATCTCTCAG**	**60**	76, 115, 191	15

### Statistical analysis

The results were expressed as mean ± standard deviation (Table 1). Numerical data were analyzed using paired student's t-test, while one way ANOVA was used to evaluate significant biochemical and molecular results. Allele and genotype frequencies were calculated and the Pearson s' chi-square (X2) test (statistical approach used to compare observed data we would expect to obtain according to a specific hypothesis) was used to determine their associations in case and control participants (BsmI and FokI) in table 3. The results were considered statistically significant when p-value < 0.05 using the Statistical Package for Social Sciences (SPSS version 16.0).

**Table 1:** Comparison of biochemical parameters between diabetic and control groups

Parameter	Diabetic subjects (n=500)	Normal subjects (n=200)	p -Value
FBS (mg/dL)	146 ± 5.54	82 ± 3.55	> 0.0001
RBS (mg/dL)	201 ± 6.74	131 ± 4.85	> 0.0001
HbA1c (%)	7.53 ±0.69	5.45 ± 0.33	> 0.0001
Vitamin D (mg/dL)	12.69 ± 1.85	21.36 ± 2.34	> 0.0001

Data expressed as mean ± SD and p value;

SD: standard deviation, n: number of subjects, FBS: fasting blood sugar, RBS: random blood sugar, HbA1c: glycated hemoglobin

**Table 3:** The genotype and allele frequencies of *Fok I* and *Bsm I* in VDR gene between T2DM patients and controls

Polymorphism	Patients n = 500(%)	Controls n = 200 (%)	OR (95% CI )	*p- Value*
***FokI*** Genotypes FF Ff ff Alleles F f	120 (24) 280 (56) 100 (20) 104 (51.7) 98 (48.3)	65(32.5) 105(52.5) 30(15.0) 226 (54.7) 186 (45.3)	1.0 (Ref) 1.64 (0.92–2.90) 1.26 (0.63–2.52) 1.00 (Ref) 1.15 (0.82–1.60)	0.080 0.513 0.443
***BsmI*** Genotypes bb bB BB Alleles b B	365(73) 120(24) 15(3) 173 (85.5) 29 (14.5)	162(81) 34(17) 4(2) 369 (89.4) 43 (10.6)	1.0 (Ref) 1.61 (0.90–2.88) 1.13 (0.20–6.30) 1.0 (Ref) 1.44 (0.87–2.38)	0.103 1.002 0.152

## Results

The genotype and allele frequencies of Fok I and Bsm I polymorphisms are shown (Table 3).

It had been clear from DNA sequencing, that F, f, B and b represented C, T, A, and G, respectively (Figure 1). Following genotyping produces by PCR-RFLP and DNA sequencing was 100% concordant. Genotype frequency distributions were in Hardy-Weinberg equilibrium in both groups calculated. The FF, Ff, and ff genotypes of FokI were 24 %, 56 %, and 20 % in T2DM patients while, 32.5 %, 52.5 %, and 15 % in controls respectively. The frequencies of F and f alleles of FokI were 51.7 % and 48.3 % in subjects and 54.7 % and 45.3 % in controls. The genotype frequencies of the bb, bB, and BB of BsmI were 73 %, 24 %, and 3 % in T2DM patients and 81 %, 17 %, and 2 % in healthy controls. The frequencies of b and B alleles of BsmI were 85.5 % and 14.5 % in cases and were 89.4 % and 10.6 % in controls. No significant associations were noted in the genotype and allele frequencies of the FokI and BsmI polymorphisms between the T2DM patients and controls (For FokI: OR = 1.15, 95% CI: 0.82–1.60; for BsmI: OR = 1.44, 95% CI: 0.87–2.38).

**Figure:**
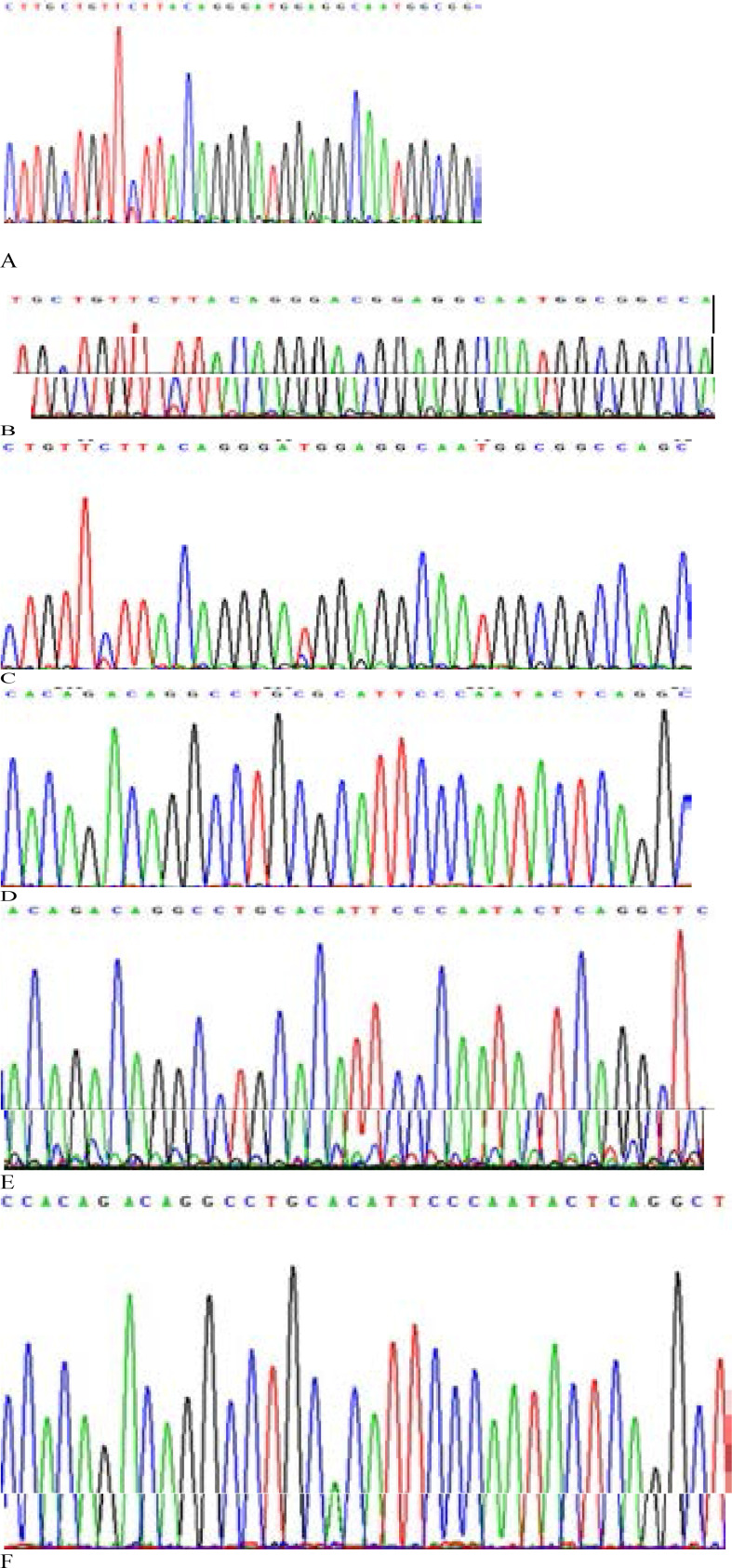


## Discussion

In addition, vitamin D receptor gene consists of many more single nucleotide polymorphisms (SNPs) other than the four demonstrated in this study. The present study was restricted to only two SNPs in the following population (BsmI, FokI,) the most examined polymorphisms. We found no significant relationship in the distribution of VDR polymorphisms between T2DM patients and healthy controls, which recommended that VDR polymorphisms may not considerably take part to the susceptibility of T2DM in the North England. The frequency of some other typical alleles located on Bsm I and Fok I had not significant difference in not alike populations, except some which were not. Similarly, the occurrence of the F allele on Fork I was 51.7%, which was not considerably same in Japanese and as well as Caucasians (53.0–60.7%) 16,17 Although, 85.5% frequency of b allele of BsmI was in English population as in Asian, Korean and Japanese (91.7%, 92.1%, and 87.4%, respectively), 18–20 while it was 52.9–57.7% in Caucasians^21^–^24^ Taken concomitantly, following data suggested that the allocation of these frequencies of VDR gene might not similar among the different ethnic groups, therefore ancillary studies on VDR polymorphisms among different racial populations would be supportive. The VDR gene located on chromosome 12q in human genome was first time cloned in 1988 and found that it contain 9 exons along 6 isoforms of exon 1 within 60–70 kb of genomic sequence^25^,^27^. It considered as important regulator of immune system interact with target cell nucleus to do a range of biochemical functions; calcium phosphorous metabolism, apoptosis and cell discrimination^28^–^30^, ^31^

Furthermore, it adjusts the effectiveness of RNA polymerase II-mediated transcription through particularly binding with active type of vitamin D3.^35^–^37^ Vitamin D3 VDR shortage consequences in various immune-mediated disorders; IBS (inflammatory bowel disease) 32.

The supplementation of vitamin D3 can change the expression of various vital organs efficacy, improve the diabetic complications and as well as reduce the risk of onset of the following disorder.

VDR gene is expressed in various tissues of the body including pancreatic tissue that play important role in synthesis of insulin and homeostasis of glucose. Present study was conducted to investigate the contribution of VDR genotypes in susceptibly of T2DM in present population.

The present study validated that no VDR gene polymorphisms were linked with the susceptibility of T2DM in English population elucidated by the differences of VDR gene variants T2DM and healthy control subjects (p< 0.005).

The variation of a single Fok I restriction site may direct to ATG start cordon modification in second exon of vitamin D receptor while infinitesimal differences on the Bsm I site can manipulate the expression of protein^33^ consequently the polymorphisms which belongs to both genes were integrated in the current study, though, no considerable link was noted between T2DM and its onset and polymorphism of VDR (FokI, BsmI). In agreement with present results, a current study has confirmed BsmI and FokI polymorphisms of VDR gene as a not possible risk factor for T2DM, Bid et al.^32^. Previously studied the relationship among four variants VDR polymorphisms with T2DM and exhibited that allele of BsmI and FokI were not significantly linked with T2DM 28–30,33.

On the conflicting, there are studies describing no relationship between type 2 diabetes mellitus patients and healthy subjects in the allele as well as genotype frequencies in vitamin D receptor FokI gene polymorphism^21^–^24^,^33^–^36^. Molecular description for the fictional association between polymorphism of FokI genotype and T2DM are only partly understood. However it is hard to decipher the exact reasons for such discrepancies, a number of possibilities should be measured; genetic trait variations, polymorphism of VDR gene is separate in specific population, different ethnicity and geographic area, T2DM is a multi-factorial disorder and different people could be bare to different geographical factors and genetic susceptibility have caused diverse results. Finally, the unsatisfactory study design may also be the reasons, like limited knowledge on non-random sampling and prospect of collection bias from the hospital based case-control study. In conclusion, it is evident that vitamin D deficiency has prevailed in said population with T2DM. Alterations in vitamin D action may affect insulin sensitivity, beta-cell function or both. Moreover our study documents that no correlation found between VDR BsmI and FokI gene polymorphisms and susceptibility to T2DM in the English population. The possible role of vitamin D in the pathogenesis of T2DM is far from being completely understood. Additionally, further knowledge on this issue may identify new candidate targets in the treatment and prevention of the disease. Therefore, further investigations on this issue are warranted.

With the submission of this manuscript, I would like to undertake that the above-mentioned manuscript has not been published elsewhere, accepted for publication elsewhere or under editorial review of publication elsewhere; and my institutes representatives are fully aware of this submission.
